# Lactation challenges of resident physicians- results of a national survey

**DOI:** 10.1186/s12884-020-03436-3

**Published:** 2020-12-09

**Authors:** Gabrielle W. Peters, Aleksandra Kuczmarska-Haas, Emma B. Holliday, Lindsay Puckett

**Affiliations:** 1 Department of Therapeutic Radiology, Yale School of Medicine, New Haven, USA; 2 Department of Human Oncology, University of Wisconsin Carbone Cancer Center, Madison, USA; 3 Department of Radiation Oncology, MD Anderson Cancer Center, 1515 Holcombe Blvd Unit 1240, Houston, TX 77030 USA; 4 Department of Radiation Oncology, Medical College of Wisconsin, Wauwatosa, USA

**Keywords:** Graduate medical education, Lactation, Support, Pumping, Maternity

## Abstract

**Background:**

There are unique challenges to parenting in residency and there is limited data to guide policy regarding lactation facilities and support for female physicians-in-training. We aimed to assess issues surrounding breast-feeding during graduate medical training for current residents or recent graduates from United States (US) residency programs.

**Methods:**

A national cross-sectional survey was sent to current and recently graduated (2017 and later) female residents in June 2020. This questionnaire was administered using the Qualtrics Survey tool and was open to each participating woman’s organization for 4 weeks. Summary statistics were used to describe characteristics of all respondents and free-text responses were reviewed to identify common themes regarding avenues for improvement.

**Results:**

Three hundred twelve women responded to the survey, representing a 15.6% response rate. The median duration of providing breastmilk was 9 months (IQR 6–12). 21% of residents reported access to usable lactation rooms within their training hospital, in which 12% reported a computer was present. 60% of lactating residents reported not having a place to store breast milk. 73% reported residency limited their ability to lactate, and 37% stopped prior to their desired goal. 40% reported their faculty and/or co-residents made them feel guilty for their decision to breastfeed, and 56% reported their difficulties with breastfeeding during residency impacted their mental health.

**Conclusion:**

Residents who become mothers during training face significant obstacles to meeting their breastfeeding/pumping needs and goals. With these barriers defined, informed policy change can be instituted to improve the lactation experience for physicians-in-training.

## Background

The number of women physicians has been rising since the 1990s. Today, women comprise over 50% of graduating medical school classes and more than one third of actively licensed doctors nationally [1, 2]. In the United States (US), 4 years of medical school is followed by 3 to 7 years of specialty specific post-graduate education, termed residency, prior to board certification. For those desiring a family, it is common to have children during residency, which typically constitutes childbearing years; the number of women electing to have children as trainees has increased in recent years [3]. Several studies have assessed perceptions of pregnancy during medical training, the availability of maternity leave, and the impact of motherhood on academic career pursuits [4–6]. However, there is little data regarding the support provided to resident physicians upon return to clinical duties after maternity leave, the feasibility of continuing lactation schedules when faced with the logistical rigors of the hospital, and complicating factors of restricted time and access to private rooms for pumping.

The American Academy of Pediatrics (AAP) recommends breastmilk as the exclusive form of nutrition for the first 6 months of an infant’s life, followed by continued breastmilk for 1 year or longer, while the World Health Organization (WHO) recommends breastfeeding for 2 years or beyond [7, 8]. For most women, this requires ~ 4–10 feedings and/or pumping sessions per day and pumping typically takes 15 to 30 min, depending on the individual. In previous specialty-specific publications, women physicians reported early cessation of breast-feeding/lactation due to insufficient time and/or privacy as well as inadequate facilities [5, 9], resulting in negative emotions and lower work satisfaction [10]. In a report of faculty/attendings across medical specialties, the lack of lactation facilities and time available for breast pumping after returning to work was the most frequently cited negative experience following childbirth [6]. Although informative, these studies have been small, single-specialty and had varying inclusion of both residents and faculty members.

The unique challenges to parenting in residency stems from the lack of autonomy, inflexibility of schedule, power discrepancy, and fear of perceived laziness or inefficiency by their superiors which may impact their long-term careers. These limit the ability for a resident physician to advocate for themselves and their family’s needs. The Accreditation Council for Graduate Medical Education (ACGME) recently instituted the requirement of a lactation room in training hospitals [11], but compliance with this policy is currently unknown. Furthermore, it is unclear whether the amount of space is sufficient, and the location of such facilities are usable and convenient for physicians-in-training.

Herein, we report the results of a national survey, which assessed issues surrounding breast-feeding in post-graduate medical training for current residents or recent graduates from United States (US) residency programs.

## Methods

This national, cross-sectional anonymous survey of residents was reviewed by the Yale institutional review board (IRB) and determined to be exempt from obtaining consent. Eligible participants included residents and fellows or recent graduates (completing residency between 2017 and 2020) from any specialty. Invitations were sent in June 2020 and were distributed to the membership email lists of several large women’s national organizations with the express permission of those organizations. Participating organizations included the American Medical Women’s Association (AMWA), Association of Women in Psychiatry (AWP), Association of Women in Surgery (AWS), American Association for Women in Radiology (AAWR), and Society of Women in Radiation Oncology (SWRO). From the five participating organizations, the estimate of total eligible participants was 2000.

### Survey instrument

The survey instrument (Supplementary Figure 1) was created through an interative process via collaboration with current residents and recent graduates and informed by prior validated questionnaires [12–14]. The survey was interactive with alternative survey paths based on participant responses (i.e., respondents without children were not exposed to the same number and scope of questions as those with children). Domains of interest included demographics, childbearing (include adoption and/or surrogacy) status, access to maternity leave, breastfeeding plans and goals, available types of resources and support upon returning to work, how lactation support (or lack thereof) impacted womens’ decision or duration of lactation, general views and opinions of pregnancy and parenthood during residency, and the health of the respondent as well as their child during the first year postpartum. The complete survey is available in supplemental material.

This questionnaire was administered using the Qualtrics Survey tool (Qualtrics, Provo, UT). The survey was open to each organization for 4 weeks.

### Data analysis

Inclusion criteria for response analysis was female gender and currently in residency in the US, or graduated from US residency since 2017, and sufficient completion of the survey. Responses from women who had graduated residency in 2016 or earlier were excluded along with respondents with incomplete demographic information and/or childbearing status. Summary statistics were used to describe characteristics of all respondents in regard to maternity leave experience and desires to breastfeed. The analysis of lactation and breastfeeding/pumping duration, availability of usable lactation facilities, and barriers to attaining lactation goals was performed for childbearing respondents. All analysis was conducted using the statistical software package SPSS, Version 26 (SPSS Inc., Chicago, Ill., USA). For the qualitative analysis, free-text responses were reviewed by the authors and common themes were identified regarding avenues for improvement in the breastfeeding experience.

## Results

Three hundred women responded to the survey, representing a 15.6% response rate. In total, 255 of these responses were eligible for analysis (*n* = 57 met exclusion criteria), and 147 of them had children during their training. Respondent characteristics are summarized in Table 1 and included a wide range of specialties, region and level of training levels, and childbearing status.

**Table 1:** Survey Respondent Characteristics

Characteristic	*N* = 255 (%)
Specialty	
Anesthesiology	1
Emergency Medicine	3
Dermatology	5
Internal Medicine	7
Obstetrics/Gynecology	3
Pediatrics	7
Psychiatry	14
PM&R	1
Radiology	51
Radiation Oncology	63
Surgery	95
Other^a^	5
Current Age	
20–25	2
26–30	60
31–35	139
36–40	48
40+	6
Current training level	
PGY1	13
PGY2	26
PGY3	36
PGY4	45
PGY5	45
PGY6	20
PGY7+	10
Graduate^b^	60
Region	
Pacific West	28
Mountain West	8
West South Central	17
West North Central	33
East South Central	21
South Atlantic	19
East North Central	61
Middle Atlantic	42
New England	26
Relationship Status	
Single	27
In a relationship	21
Married	203
Separated/Divorced	3
Widowed	1
Number of Children	
0	108
1	109
2	36
3	2

^a^Other includes transitional year (2), fellowship (1), and combination programs (medicine/pediatrics (1) and medicine/psychiatry (1)). ^b^Includes graduates from 2017 to 2019. PM&R = physical medicine and rehabilitation

Of the women who became parents during training, 98.6% (145/147) took some form of maternity leave for a median of 6 weeks (IQR 6–9) with various combinations of FMLA, research/elective, and vacation time. Ten women (6.8%) reported having to extend residency due to maternity leave.

### Lactation goals

All women who had children during residency reported intending to breastfeed during maternity leave and 97% (138/142) intended to continue providing breast milk through pumping upon return to clinical duty. Eighty-seven percent (123/142) and 27% (52/142) intended to breastfeed past six and 12 months, respectively. The median total time of breastfeeding and pumping was 9 months (IQR 6–12 months), with 74% of respondents continuing after 6 months, and 13% continuing past 12 months. After excluding sixteen women who were currently still breastfeeding, the median total lactation period was 11 months, IQR 6–12.

### Lactation facilities

**Fig. 1:**
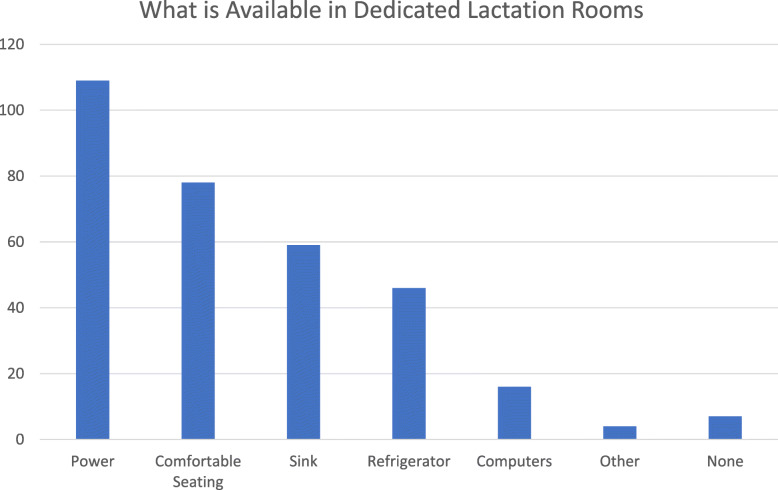
Resources available in dedicated lactation rooms displayed in frequency. One hundred forty seven residents responded to this question by selecting all resources available in dedicated lactation rooms at their institution. Multiple selections were allowed

**Fig. 2:**
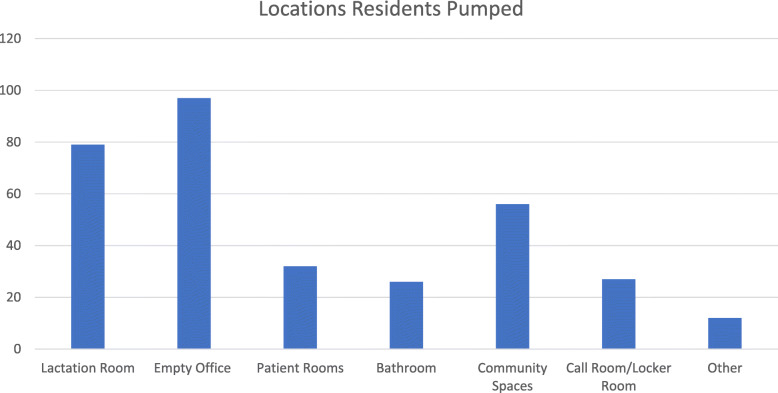
Locations utilized for breastmilk expression displayed in frequency. One hundred forty seven residents responded to this question by selecting all places they used as lactation rooms. Multiple selections were allowed

Ninety-one percent of residents (124/136) reported the presence of lactation rooms in their training hospital. However, only 21% (33/124) reported the rooms to be usable with the remainder being inaccessible due to location or always being occupied when needed. Thirty-eight percent (51/126) of residents reported lactation rooms within their home departments, and of these, 51% (26/51) were reported to be accessible. When present, dedicated lactation rooms commonly had power outlets and comfortable seating (80 and 67%, respectively), but less than half provided a sink (43%) or refrigerator (33%), and only 12% had a computer. (Fig. 1) Sixty percent of residents reported not having a place to store breastmilk. Other locations used by residents for pumping are shown in Fig. 2. The majority of responding residents reported it typically took them 10–15 min to find a place to pump.

### Barriers to lactation

Seventy-three percent of respondents (98/134) reported residency limited their ability to lactate; 61% noted diminished supply due to insufficient time/locations to pump, and stress. Other limiting factors included lack of support from program (16%) and co-residents (10.5%). Ninety-nine percent of respondents (126/127) experienced complications from inadequate pumping, including 64% with diminished supply, 26% with clogged ducts, and 16% mastitis. Overall, 37% (50/136) stopped pumping prior to their desired goal, and 56% (74/133) reported their inability to breastfeed during residency impacted their mental health. Forty-eight percent of lactating residents (62/132) reported their faculty and program leadership made them feel guilty for pumping, predominantly due to the time spent out of clinic or procedures and perceived accommodations. Forty percent (53/132) reported their co-residents made them feel guilty for the same reasons.

### Steps for improvement

Respondents were asked what may have improved their lactation experience during residency, and 50% (127/255) provided optional free text responses.

The most common theme, identified 55 times, included having more dedicated lactation rooms which were clean, private and fully equipped (including computers, sinks, outlets and breast milk storage) in accessible locations. On this topic, one respondent stated that it would help if the “*designated lactation room (was) in my place of work- i.e. the clinic building- rather than across the street in the basement of the hospital.*” Another respondent said that there were only “*7 lactation rooms for all general staff in the entire hospital- approximately 300 lactating women. Completely insufficient resources, also no place to store pumped milk.*” Several women also mentioned receiving complaints or criticism when pumping in shared workspaces. One woman stated *“(one) faculty told me I was gone too much from the reading room and (another) telling me that pumping at my workstation while on call was “disgusting”.*”

A theme mentioned 40 times was the need for more dedicated time, scheduled breaks, and flexibility within their clinical schedule. One respondent recommended “*15 minutes free every four hours*”, another recommended “*protected and designated time twice a day to pump*”, and another recommended “*automatically adding in 20-minute breaks in the clinic schedule*”. Convenient space and sufficient time were of particular concern to women in surgical residencies as lactation spaces, when available, were often not close enough to the operating room. One woman said it would help to have the “*availability of time; it was hard to do it in between surgical cases, while peeing, eating, drinking enough water and getting the next patient ready.*”

Another common suggestion was clear policies and expectations guiding attendings and co-residents of the complications and difficulties which are associated with inadequate pumping, mentioned 49 times. One woman recommended “*having a written policy for the department*”. Another mentioned that it would help to have “*clear, mandatory expectations for my all-male, childless, less-than-happy about my ‘family needs’ faculty*”. One woman eloquently offered advice to programs to support lactation: “*breastfeeding is evidenced based. It should be mandatory that programs and faculty not only support this practice but expect it and therefore plan for it. No woman should ever be made to feel that she is not allowed to provide food for her child.*”

Many expressed regrets for not advocating for themselves more and asking for help in clinic, on call or in the operating room. Finally, several women mentioned what would help most would be to “*normalize breastfeeding*” and have more female leaders, attendings and colleagues to set a greater precedent. Additionally, several women mentioned that having increased paid maternity leave would help lactation success. One woman mentioned “*maternity leave policies are atrocious in the US especially in surgical programs and need to be reformed at the ABMS [American Board of Medical Specialties] and ACGME levels”.*

## Discussion

In this national survey study of current residents and recent graduates, we found that women who become mothers during residency training face many obstacles to meeting their breastfeeding and pumping goals upon returning to clinical duty. Most commonly cited challenges included the lack of convenient, accessible space for pumping breastmilk. There was much perceived pressure from program leadership, attendings, and co-residents due to the time and accommodations required to pump. Nearly all surveyed residents reported experiencing at least one lactation complication thought to be due to inadequate pumping. Despite these obstacles, nearly three quarters were able to provide breastmilk to their infant for more than 6 months.

Prior specialty-specific publications on physician lactation while comparatively small or focused predominantly on attending physicians, also demonstrate an approximate 40% rate of early breast-feeding/lactation cessation, with 15–25% continuing to provide breastmilk past 1 year [5, 9, 10]. A majority of women reported insufficient lactation facilities [5, 6, 15], and a quarter experienced workplace discrimination [6]. In these studies, early cessation of breastfeeding was associated with negative emotions and poor work satisfaction [9, 10]. According to the 2020 US National *Healthy People* objectives, the CDC aims for 36% of mothers to provide breastmilk past 1 year, which is nearly three times the current rate attained by female US resident physicians [16]. In Canada, half of lactating residents felt unsupported by their academic institutions and/or residency association regarding labor rights or pumping needs, and 30% felt penalized and/or criticized. Similar to our analysis, a third of residents did not meet personal breastfeeding goals with the majority reporting inadequate time and space. Using the same definition for lactation-related complications, approximately half of lactating residents experienced a complication, compared to 99% in our sample [13].

As part of the 2010 Affordable Care Act (ACA), employers are mandated to provide an on-site private space, other than a bathroom, shielded from view and free from intrusion in addition to “a reasonable amount” of breaktime for mothers to express breast milk, continuing 1 year after the child’s birth [17] for employees subject to overtime pay;, however, resident physicians are not included in this group. As of 2018, the ACGME added lactation facilities to their list of common program requirements, stipulating presence of a clean and private lactation room within the sponsoring institution with refrigeration capability and “proximity appropriate to patient care.” [11] While the advantage of access to a computer for continued work while pumping is recognized in the document, its presence is not mandatory. Notably, this does not guide the number of rooms, is vague regarding appropriate proximity, is not department-specific, and references the sponsoring institution alone. This is particularly problematic for residents in outpatient facilities, those rotating at satellite hospitals, and those in surgical specialties.

Resident physicians face additional barriers to breastfeeding compared to their attending counterparts [18], and are uniquely limited in their ability to advocate for themselves due to lack of autonomy and significant power discrepancy. Embedded within the universally identified hierarchy of medicine is the threat of resident intimidation, humiliation, and influence on one’s future career prospects. For these reasons, barriers must be defined and addressed with specific interventions.

To combat the obstacles noted above, the ACGME minimally acceptable provisions for lactating residents need to be expanded. This should include mandating multiple dedicated resident-only lactation rooms based on an institution’s number of potentially lactating resident physicians. These must be dispersed throughout the primary training institution and at any site residents will be rotating, with facilities specifically present near operating/procedure rooms, and within outpatient centers. The stipulation of providing refrigeration for breast milk within lactation rooms needs to be mandated and computer access added as a requirement. A clear ACGME policy regarding a minimum frequency of protected time for lactating women may lessen the burden residents feel when asking their attendings to be excused for pumping; for example, 20 to 30-min breaks every 3 h plus transit time [19]. In response to the commonly shared stigma inciting feelings of guilt, institutions should consider adding mandatory education for all faculty and residents regarding the medical necessity for lactating women to be provided adequate time to express breast milk in order to avoid health complications. This also aligns with the Surgeon General’s call to action for improving breastfeeding in the US [20].

While our analysis is unique in both its scope and depth, there are some limitations. Not all medical specialties were evenly represented. Although investigators endeavored to include more specialty organizations, lack of resident and/or early career female physician only listserves prohibited this. While survey questions were carefully worded to allow for interpretation of residency impact on decision to stop lactating prior to initially intended timeframe, the cross-sectional nature of the study prevents establishment of direct causation. Our survey is at risk for non-response bias, with a survey completion rate of ~ 15.6%. However, it is likely that our denominator of 2000 eligible participants is an overestimation as the five organizational list-serves were heterogeneous in their makeup, including some medical students and graduates prior to 2017.

## Conclusion

Our data show that resident physicians who become mothers during training are resilient and resourceful, but their relative success in lactation comes at the expense of their emotional well-being, undue stress, and frequently, medical complications. Systemic changes are necessary, and we encourage national GME policy change while recognizing this will take time. In the interim, we encourage program directors (PDs) and institutional GME offices to move forward by anticipating the challenges faced by resident physicians, providing pre-partum mentorship, and planning for necessary space/resource allocation. Proactive identification of these facilities and resources for use and planning for the transition period will promote a sense of support and autonomy. PDs should also develop and disseminate a minimum frequency of protected time to be excused for pumping to decrease the burden of resident requests/explanation for these sessions.

## Supplementary Information

**Additional file 1.**

## Data Availability

The datasets generated and/or analyzed during the current study are not publicly available out of respect for survey respondent privacy and the sensitive/personal nature of survey questions, but anonymous data are available from the corresponding author on reasonable request.

## Abbreviations

AAP: American Academy of Pediatrics
WHO: World Health Organization
ACGME: Accreditation Council for Graduate Medical Education
IRB: Institutional Review Board
AMWA: American Medical Women’s Association
AWP: American Women in Psychiatry
AWS: Association of Women in Surgery
AAWR: American Association of Women in Radiology
SWRO: Society of Women in Radiation Oncology
IQR: Interquartile range
FMLA: Family and Medical Leave Act
US: United States
CDC: Centers for Disease Control
ACA: Affordable Care Act
PD: Program Director
GME: Graduate Medical Education
