# Supplementary material for: Lactation challenges of resident physicians- results of a national survey
Source: BMC Pregnancy Childbirth. 2020 Dec 9;20:762. doi: 10.1186/s12884-020-03436-3 (PMC7724857; doi:10.1186/s12884-020-03436-3)
Supplement: Supplementary file 1 — Additional file 1. [file 12884_2020_3436_MOESM1_ESM.pdf]

Demographics

What is your age?

- ○ <20
- ○ 20-25
- ○ 26-30
- ○ 31-35
- ○ 36-40
- ○ 40+

What specialty are you training in/did you recently graduate from?

- ○ General Medicine (Internal Medicine and Family Practice)
- ○ Pediatrics
- ○ OB/GYN
- ○ Emergency Medicine
- ○ Anesthesiology
- ○ Surgery (including orthopaedic, urology, opthalmology, vascular, otolaryngology, neurosurgery)
- ○ Psychiatry
- ○ Neurology
- ○ Radiology
- ○ Radiation Oncology
- ○ PM&R
- ○ Other ____________

What is your current level of training

- ◯ PGY1
- ◯ PGY2
- ◯ PGY3
- ◯ PGY4
- ◯ PGY5
- ◯ PGY6
- ◯ PGY7
- ◯ 2017-2019 residency graduate
- ◯ Other ____________

What year did you graduate residency?

- ◯ 2017
- ◯ 2018
- ◯ 2019

In what country are you (or did you complete) training?

- ◯ United States
- ◯ Other ____________

If you are training/trained in the United States, what region was your program in? (see map https://www2.census.gov/geo/pdfs/maps-data/maps/reference/us_regdiv.pdf)

- ◯ Pacific West
- ◯ Mountain West

- West South Central
- West North Central
- East South Central
- South Atlantic
- East North Central
- Middle Atlantic
- New England

What is your current marital status?

- Single
- Married
- Divorced
- Widowed
- In a relationship
- Separated
- Other

To your knowledge, does your residency program/institution currently have a maternity leave policy in place?

- Yes, separate from vacation/elective time and FMLA
- In the form of FMLA
- No
- I'm not sure
- Other ________

When did you become aware of the maternity leave policy?

- ○ When I interviewed for residency
- ○ During residency orientation
- ○ When a colleague became pregnant
- ○ When I became pregnant
- ○ Other ____________

Did you have a child (whether through birth or adoption) during residency?

- ○ Yes
- ○ No

How many children were born or adopted by you during your residency training?

____

Did you take maternity leave during residency (whether for biologic, adoptive/surrogate, or foster children)? (check all that apply)

- ☐ I took maternity leave in the form of FMLA
- ☐ I took maternity leave in the form of vacation
- ☐ I took maternity leave in the form of elective/research time
- ☐ No
- ☐ Other ____________

How many weeks of maternity leave did you take? (If you have had multiple children during residency, report with respect to your most recent child)

____

How many of your maternity weeks were vacation or elective time? (If you have had multiple children during residency, report with respect to your most recent child)

☐ ____________________

How many weeks will you need to extend residency because of your maternity leave?

- ○ Extended by (w) ____________
- ○ N/A

Did your coresidents ever make you feel guilty regarding your decision to have children (**check all that apply**)?

- ☐ Yes, due to increased workload on them during pregnancy
- ☐ Yes, due to increased workload on them during maternity leave
- ☐ Yes, due to the extra time or days missed for daycare closings or kids being sick
- ☐ No, I felt supported
- ☐ Other ____________

Did your faculty and/or program director ever make you feel guilty regarding your decision to have children (**check all that apply**)?

- ☐ Yes, due to increased workload on them during pregnancy
- ☐ Yes, due to increased workload on them during maternity leave
- ☐ Yes, due to the extra time or days missed for daycare closings or kids being sick
- ☐ No, I felt supported
- ☐ Other ____________

Did you <u>intend</u> to breast-feed while on maternity leave? (If you have had multiple children during residency, report with respect to your most recent child)

- ◯ Yes
- ◯ No

Did you <u>intend</u> to breast-feed after you returned to clinical duties? (If you have had multiple children during residency, report with respect to your most recent child)

- ◯ Yes
- ◯ No

How long did you <u>intend</u> to breast-feed total (**in months**)? (If you have had multiple children during residency, report with respect to your most recent child)

- ◯ Up to 4 months
- ◯ 4-6 months
- ◯ 7-12 months
- ◯ >12 months
- ◯ Other ______

Did you breastfeed after returning to work (for any period of time)? (If you have had multiple children during residency, report with respect to your most recent child)

- ◯ Yes
- ◯ No

Did you pump after returning to work (for any period of time)? (If you have had multiple children during residency, report with respect to your most recent child)

- ○ Yes
- ○ No

Did your coresidents ever make you feel uncomfortable regarding your decision to pump/breastfeed while at work? (**check all that apply**)

- ☐ Yes, due to time required out of the office/out of clinic
- ☐ Yes, because of special accommodations required to pump
- ☐ No, I felt supported
- ☐ Other ____________

Did your faculty and/or program director ever make you feel uncomfortable regarding your decision to pump/breastfeed while at work? (**check all that apply**)

- ☐ Yes, due to time required out of the office/out of clinic
- ☐ Yes, because of special accommodations required to pump
- ☐ No, I felt supported
- ☐ Other ____________

How long did you breast-feed/pump milk for your child/children during residency total (**in months**)? (If you have had multiple children during residency, report with respect to your most recent child)

____

Were/are there dedicated lactation rooms available within your residency department?

- ◯ Yes and they were typically available when I needed them
- ◯ Yes but they were often full
- ◯ Yes but they were in an inconvenient location
- ◯ No
- ◯ Unsure

Were/are there dedicated lactation rooms available at your residency training hospital?

- ◯ Yes and they were available when I needed them
- ◯ Yes but they were often full
- ◯ Yes but they were in an inconvenient location
- ◯ No
- ◯ Unsure

In lactation rooms, were the following resources available **(check all that apply)**?

- ☐ Power outlets
- ☐ Computer
- ☐ Comfortable seating
- ☐ Sink
- ☐ Refrigerator
- ☐ Other ______
- ☐ None of these were available

Where did you pump? (**check all that apply**)

- ☐ Lactation room
- ☐ Empty office
- ☐ Patient Rooms
- ☐ Bathrooms
- ☐ Community work spaces
- ☐ Other (specify) ____________
- ☐ N/A

On average, how much time did searching for a location to lactate take away from clinical duties (each time)?

- ◯ <10 minutes
- ◯ 10-15 minutes
- ◯ 15-20 minutes
- ◯ 20-30 minutes
- ◯ > 30 minutes
- ◯ N/A

Did you experience complications perceived to be related to inadequate pumping, such as mastitis, clogged milk ducts, or diminished milk supply after returning to clinical duties? (**Check all that apply**; if you have had multiple children during residency, report with respect to your most recent child)

- ☐ None
- ☐ Mastitis
- ☐ Clogged milk ducts
- ☐ Diminished milk supply

☐ Other [ ]

Was there a designated place to store/refrigerate breast milk during the workday at your facility?

○ Yes

○ No

Did you stop breastfeeding earlier than planned during residency? (If you have had multiple children during residency, report with respect to your most recent child)

○ Yes

○ No

What contributed to your decision to stop pumping prior to your personal goal **(check all that apply)**?

☐ Inadequate time to pump during the day

☐ Inadequate locations to pump during the day

☐ Diminished supply of breast milk

☐ Pumping was leading me to fall behind on work, ultimately leading to longer hours

☐ I stopped for other reasons (specify) [ ]

What were the barriers to adequate time for pumping **(check all that apply)**?

☐ Procedures

☐ Floor management

☐ Clinic visits

☐ N/A

☐ Other ______

Do you feel residency limited your ability to breast-feed/pump **(check all that apply)**?

☐ Yes, due to lack of support from program

☐ Yes, due to lack of support from co-residents

☐ Yes, I believe my milk supply decreased due to stress and/or due to insufficient pumping time/frequency

☐ No, I don't feel that residency limited my ability to breast-feed

☐ Other ______

Do you regret your decision to (or not to) breast-feed?

○ Yes

○ No

○ N/A

What do you feel could have improved your ability to breast-feed after returning to work?

______

Did you have "baby blues", a depressed mood, or postpartum depression after giving birth in residency? (If you have had multiple children during residency, report with respect to your most recent child)

○ Yes

○ No

○ Unsure

Did you take time off, outside of any maternity leave used, due to mental health concerns or did your facility allow additional time off if needed (non-vacation days)? (If you have had multiple children during residency, report with respect to your most recent child)

- ◯ Yes, up to a week
- ◯ Yes, greater than a week
- ◯ No, there was no time/personal days available
- ◯ No, I did not take additional days off and am unaware if I could have
- ◯ I did not explore this option.

Do you feel your mental health negatively impacted your ability to care for patients, family or yourself?

- ◯ Patients/clinical care may have been impacted
- ◯ My family may have been impacted
- ◯ My self care may have been impacted
- ◯ No

Did you have mental health support from your program?

- ◯ Yes
- ◯ No

Which of the following interventions were helpful (**check all that apply**)?

- ◯ Passing of time and recovering from post-partum weeks
- ◯ Speaking to a therapist
- ◯ Medication from PCP or psychiatrist

- ○ Supportive peers
- ○ Exercise
- ○ Other ____________

Do you feel that your ability to breastfeed during residency impacted your mental health?

- ○ Yes
- ○ No

Did your child have health complications, illness, or growth problems in the first year (**check all that apply**)? (If you have had multiple children during residency, report with respect to your most recent child)

- ☐ Premature birth <=36 weeks
- ☐ Infant Allergy
- ☐ Failure to thrive
- ☐ >5 hospital admissions
- ☐ Otitis media
- ☐ Lower respiratory tract infections
- ☐ Necrotizing enterocolitis
- ☐ Sudden Infant Death Syndrome (SIDS)
- ☐ Urinary Tract infection
- ☐ Neonatal sepsis
- ☐ Other ____________
- ☐ No

If you have not, do you plan to parent children during residency?

- ◯ Definitely yes
- ◯ Probably yes
- ◯ Might or might not
- ◯ Probably not
- ◯ Definitely not
- ◯ N/A, Already graduated from residency

Do you intend to breastfeed when you have children (whether in residency or in practice)?

- ◯ Definitely yes
- ◯ Probably yes
- ◯ Might or might not
- ◯ Probably not
- ◯ Definitely not
- ◯ Not applicable

For those who have not had children, how long do you intend to breastfeed your children? (months)

Are you aware of lactation rooms and support at your residency institution?

- ◯ Yes
- ◯ No, but I know where to find the information
- ◯ No, and I don't know where to find the information

To the best of your knowledge, what is available in the lactation rooms at your institution (**check all that apply)**?

- ☐ Power outlets
- ☐ Computer
- ☐ Comfortable seating
- ☐ Sink
- ☐ Refrigerator
- ☐ Other ____________

What other comments or considerations do you have regarding these topics?

____

Powered by Qualtrics
